# Over Expression of Mango *MiGA2ox12* in Tobacco Reduced Plant Height by Reducing GA_1_ and GA_4_ Content

**DOI:** 10.3390/ijms252212109

**Published:** 2024-11-11

**Authors:** Yu Zhang, Ji Zhang, Guodi Huang, Yiwei Tan, Lei Ning, Mu Li, Yonglong Mo

**Affiliations:** 1 Guangxi Subtropical Crops Research Institute, Guangxi Academy of Agricultural Sciences, Nanning 530001, China; gxrzshgd@gxaas.net (G.H.); gxrzstyw@gxaas.net (Y.T.); ninglei@gxaas.net (L.N.); gxrzslm931@gxaas.net (M.L.); gxrzsmyl@gxaas.net (Y.M.); 2 Guangxi Zhuang Autonomous Region Engineering Research Center of Green and Efficient Development for Mango Industry, Nanning 530001, China

**Keywords:** mango, GA2ox, plant height, GA

## Abstract

The regulation of gibberellic acid 2-oxidase (*GA2ox*) gene expression represents a critical mechanism in the modulation of endogenous gibberellic acids (GAs) levels, thereby exerting an influence on plant height. In this context, we conducted a comprehensive genome-wide analysis of the GA2ox gene family in mango (*Mangifera indica* L.), a species of significant economic importance, with the aim of identifying potential candidate genes for mango dwarf breeding. Our findings delineated the presence of at least 14 members within the *MiGA2ox* gene family in the mango genome, which were further categorized into three subfamilies: C_19_-GA2ox-I, C_19_-GA2ox-II, and C_20_-GA2ox-I. Notably, *MiGA2ox12,* a member of the C_19_-GA2ox-II subfamily, exhibited substantial expression across various tissues, including roots, bark, leaves, and flowers. Through overexpression of the *MiGA2ox12* gene in tobacco, a distinct dwarf phenotype was observed alongside reduced levels of GA_1_ and GA_4_, while the knockout line exhibited contrasting traits. This provides evidence suggesting that *MiGA2ox12* may exert control over plant height by modulating GA content. Consequently, the *MiGA2ox12* gene emerges as a promising candidate for facilitating advancements in mango dwarfing techniques.

## 1. Introduction

Mango (*Mangifera indica* L.) is a highly esteemed tropical fruit renowned for its unique flavor and rich nutritional composition. Guangxi Province is the most significant mango-producing region in China, with Baise City alone producing over 1.23 million tons of mangoes annually. The implementation of dwarf cultivation techniques has enabled the growth of smaller and more manageable trees, which not only offer cost-effectiveness but also exhibit desirable traits such as early fruiting, high yields, and consistent production. Consequently, dwarf cultivation has emerged as a major trend in the mango industry. Research has established a strong correlation between plant height and gibberellin (GA) synthesis and metabolism.

The biosynthesis of GAs involves multiple intermediates and is a highly complex process, forming different forms of GAs that are named based on the time of discovery. Among these, GA_1_, GA_3_, GA_4_, and GA_7_ exhibit the highest biological activity and are commonly referred to as active GAs. Based on the number of carbon (C) atoms in their structures, GAs can be categorized into two main groups: C_20_-GAs (including GA_12_, GA_15_, GA_24_, GA_53_, GA_44_, GA_19_, etc.) and C_19_-GAs (including GA_9_, GA_20_, GA_1_, GA_4_, etc.). Maintaining GA homeostasis is essential for optimizing plant growth and development; hence, extensive research on GA biosynthesis has been conducted across various species, catalyzed by several key enzymes [1]. The most biologically active GA_1_ and GA_4_ undergo 2β-hydroxylation to transform into biologically less active GA_8_ and GA_34_, a reaction catalyzed by GA 2-oxidase (GA2ox) [2,3,4], which serves as a primary mechanism for regulating GA activity in plants.

GA2ox proteins are classified into C_20_-GA2ox or C_19_-GA2ox based on their substrate affinity. As members of the 2-oxoglutarate-dependent (2OG-Fe(II)) dioxygenase superfamily [4], GA2ox enzymes are identified in diverse plant species, including Arabidopsis [5,6], rice [7], wheat [8], barley [9], tomato [10], pineapple [11], and peach [12]. Overexpression of GA2ox genes has been shown to significantly reduce levels of GA_1_ and GA_4_, resulting in a dwarf phenotype [13]. For instance, ectopic expression of the *PcGA2ox1* gene from soybean in wheat markedly decreases GA content, leading to reduced plant height [14]. In rice, overexpression of *OsGA2ox1* and *OsGA2ox6* causes a substantial plant height reduction [7,15], while *OsGA2ox9* results in a moderate decrease [16]. These findings suggest the presence of a key gene within the GA2ox family that acts as an inhibitor of plant height. In addition, OsGA2ox5 and OsGA2ox8 can be expressed in the cell nucleus, which can inhibit plant height [15,17]. These two genes are also involved in regulating salt stress and osmotic stress, respectively. Therefore, the localization of GA2ox may be closely related to its function. Overexpressing representative genes from the peach GA2ox family in tobacco, specifically *PpGA2ox1*, *PpGA2ox5*, and *PpGA2ox2*, leads to reduced plant height and smaller leaf size [12]. Additionally, exogenous GA_1_ and GA_3_ can partially or fully reverse the transgenic phenotype. Overexpression of the *BnGA2ox6* gene from rapeseed in Arabidopsis induces characteristic symptoms of GA deficiency, including inhibited elongation of the lower stem, delayed seed germination, and postponed flowering [18]. Similarly, overexpression of *MdGA2ox7* in Arabidopsis results in reduced GA activity, manifesting as dwarfism and delayed flowering, with effects that can be alleviated through the application of exogenous GA_3_ [19]. These findings collectively highlight the ability of GA2ox family genes to modulate plant height by decreasing GA levels.

However, the GA2ox family in mangoes has not been thoroughly investigated. In this study, we identified 14 *MiGA2ox* genes in the mango genome and analyzed their gene and protein structures, protein motifs, chromosome distribution, and expression profiles. Phylogenetic analysis categorized these genes into three subgroups: C_19_-GA2ox-I, C_19_-GA2ox-II, and C_20_-GA2ox-I. Notably, *MiGA2ox12*, a member of the C_19_-GA2ox-II subgroup, exhibited high expression levels in the root, bark, leaf, and flower. Functional assessment through overexpression in tobacco revealed that these lines displayed a dwarf phenotype alongside reduced levels of GA_1_ and GA_4_. As expected, the results of gene editing and overexpression lines were opposite to what was anticipated. Our findings provide a comprehensive overview of the GA2ox gene family in mango, paving the way for further functional analyses and potential improvements in mango variety enhancement through gene transfer methods.

## 2. Results

### 2.1. Identification and Characterization of MiGA2ox Proteins in Mango

**Figure 1:**
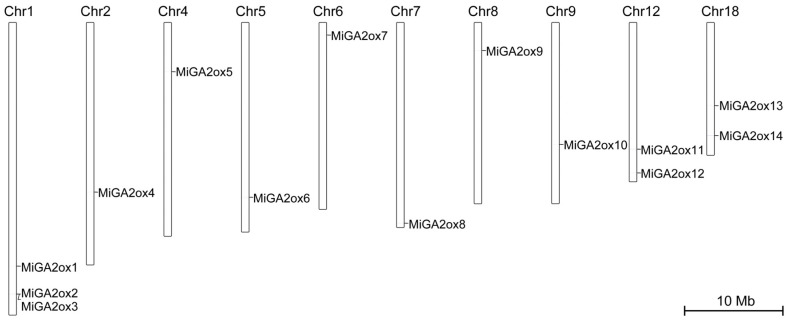
Chromosomal localization of *MiGA2ox* genes.

**Figure 2:**
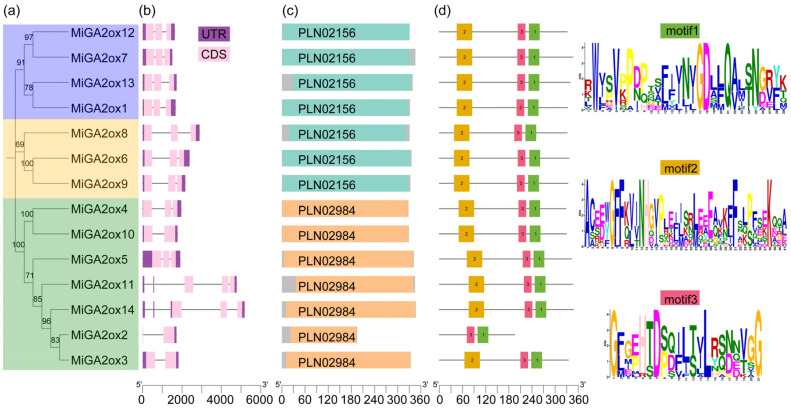
Identification and bioinformatics analysis of the MiGA2ox gene family. (**a**) Phylogenetic tree based on Maximum Likelihood (ML) method, showing the relationships among 14 MiGA2ox proteins. The tree was constructed using the MEGA 5.0 program and subjected to 1000-times bootstrap resampling. (**b**) Structure analysis of *MiGA2ox* genes. Coding sequences (CDS) are indicated by pink boxes, while introns are represented by black solid lines. Upstream or downstream untranslated regions (UTRs) of the genes are shown in purple boxes. (**c**) Conserved domain and (**d**) conserved motifs analysis of the MiGA2ox proteins, with different domains and motifs highlighted in different-colored boxes.

In the study, a Basic Local Alignment Search Tool (BLAST) search was performed using eight gibberellic acid 2-oxidase sequences from *Arabidopsis thaliana* (AtGA2oxs) as queries to identify candidate GA2ox proteins in mango (*Mangifera indica* L.) by comparing them against the mango genome. Subsequently, motif analysis was employed to confirm the identity of the candidate MiGA2ox proteins. A total of 14 MiGA2ox proteins were ultimately identified (Table 1). The naming convention of these MiGA2ox proteins was determined based on their chromosomal locations (Table 1 and Figure 1). The number of exons within the MiGA2ox genes ranged from 1 to 3 (Figure 2b), with amino acids (AA) counts varying between 195 and 347. Moreover, the molecular weights (MWs) of these proteins ranged from 21.99 to 39.96 kDa, and the isoelectric points (PIs) fell between 5.31 and 7.7 (Table 1). These MiGA2ox proteins exhibited conserved domains (PLN02156 or PLN02984) and unique motifs, enabling their classification into three distinct groups (Figure 2a,c,d).

### 2.2. Evolutionary Analysis of MiGA2ox Proteins

**Figure 3:**
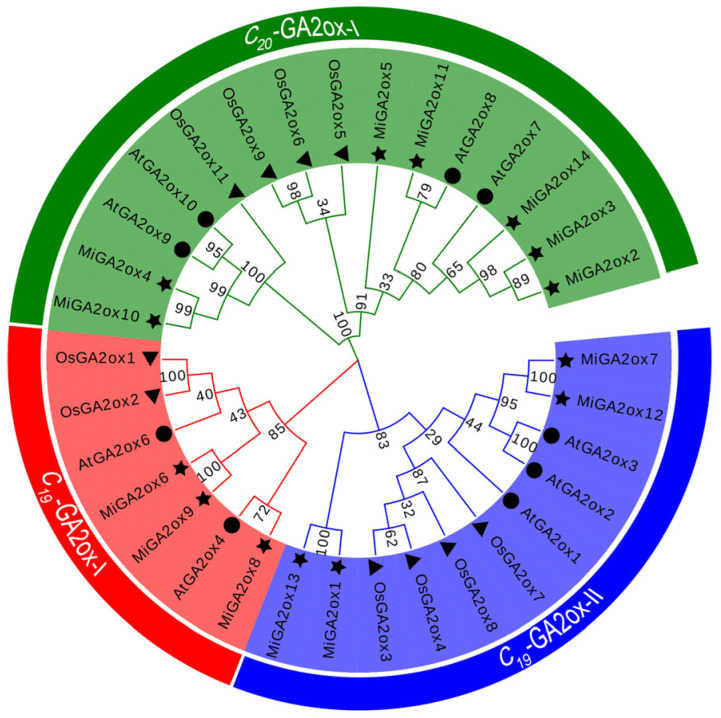
The ML phylogenetic analysis of the GA2ox protein family from mango, rice, and Arabidopsis, denoted with stars, triangles, and circles, respectively. The GA2ox proteins were categorized into three distinct subfamilies, each represented by a different background color. This phylogenetic tree was generated using the MEGA 5.0 software with 1000 bootstrap replications.

In order to comprehensively understand the evolutionary relationships, a phylogenetic tree was constructed using the Maximum Likelihood (ML) method with GA2ox proteins from mango, Arabidopsis, and rice (Figure 3). Based on their respective substrates, GA2ox proteins were categorized into three distinct subgroups, namely C_19_-GA2oxI, C_19_-GA2ox-II, and C_20_-GA2ox-I. Reports suggest that members of C_19_-GA2ox-I and C_19_-GA2ox-II exhibit activity towards C_19_-GAs, while members of the C_20_-GA2ox-I subgroup primarily demonstrate activity against C_20_-GAs. Specifically, MiGA2ox6, MiGA2ox8, and MiGA2ox9 were classified into the C_19_-GA2ox-I subgroup. MiGA2ox1, MiGA2ox7, MiGA2ox12, and MiGA2ox13 belonged to the C_19_-GA2ox-II subgroup. MiGA2ox2, MiGA2ox3, MiGA2ox4, MiGA2ox5, MiGA2ox10, MiGA2ox11, and MiGA2ox14 were grouped into the C_20_-GA2ox-I subgroup. This clustering pattern indicated the potential activities of the MiGA2ox proteins, where MiGA2ox1, MiGA2ox6, MiGA2ox7, MiGA2ox8, MiGA2ox9, MiGA2ox12, and MiGA2ox13 might be active against C_19_-GAs while, MiGA2ox2, MiGA2ox3, MiGA2ox4, MiGA2ox5, MiGA2ox10, MiGA2ox11, and MiGA2ox14 might be active towards C_20_-GAs.

### 2.3. Cis-Acting Elements and Transcription Factor Binding Sites in MiGA2ox Promoters

**Figure 4:**
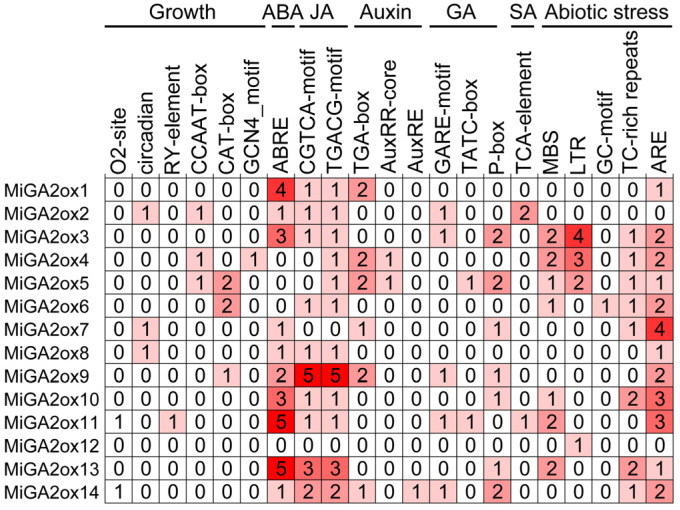
The *cis*-acting elements present in the promoters of *MiGAoxs*. The numbers represented the quantity of elements, and the varying shades of red indicated their abundance levels.

*Cis*-acting elements are essential for gene transcription and expression, providing diverse regulatory functions that influence plant growth and environment adaptation. In order to elucidate the characteristics of MiGA2ox genes and predict their potential regulatory pathways, we analyzed the types and abundance of elements within the promoter sequences of *MiGA2oxs*. Our focus was primarily on *cis*-acting elements associated with growth and development, hormonal regulation, and abiotic stress responses (Figure 4). *Cis*-acting elements linked to growth encompassed O2-site, circadian, RY-element, CCAAT-box, CAT-box, and GCN4_motif. Hormone-responsive elements included those responsive to abscisic acid (ABRE), jasmonic acid (CGTCA-motif, TGACG-motif), auxin (TGA-element, AuxRR-core, AuxRE), gibberellin (GARE-motif, TATC-box), and salicylic acid (TCA-element). Additionally, elements related to abiotic stress such as MBS, LTR, GC-motif, TC-rich repeats, and ARE were identified. Notable, ABA-responsive elements were the most prevalent. The promoters of *MiGAox1*, *MiGAox4*, *MiGAox6*, *MiGAox8*, and *MiGAox12* did not contain gibberellin response elements. But the presence of various *cis*-acting elements within the promoter regions indicated that *MiGAoxs* played important roles in regulating mango growth and development, as well as in responding to stress and hormonal signals.

Transcription factor binding sites (TFBSs) serve as regulatory elements of gene promoters. Genes that are conserved across species typically exhibit conserved TFBSs as well. We utilized the online tool PlantRegMap to predict TFBSs within the 2.0 kb promoter regions of all *MiGA2oxs*, aiming to explore their potential biological functions. Our findings revealed substantial variability in the types and quantities of TFBSs among the *MiGA2ox* gene promoters. In total, we identified 6198 TFBSs corresponding to 595 TFs within the promoter regions of *MiGA2ox* genes (Table 2). Among the 14 *MiGA2ox* genes analyzed, *MiGA2ox3* and *MiGA2ox9* possessed the highest diversity with 36 distinct TFBSs, while *MiGA2ox2* and *MiGA2ox8* only exhibited predictions for 28 TFBSs. Important transcription factors associated with plant growth and development, such as ARF, NAC, and bHLH, were found to bind to multiple sites. The diversity of these TFBSs may offer insights into how plants regulate gibberellin synthesis and metabolism through the spatiotemporal expression modulation of *MiGA2oxs*, thereby influencing growth and development. Furthermore, TFBSs involved in various abiotic stress responses, like MYB, ERF, and WRKY, were also detected in some *MiGA2ox* promoters, suggesting a potential role for *MiGA2ox* genes in mediating responses to diverse abiotic stresses.

### 2.4. Expression of the MiGA2ox Genes

**Figure 5:**
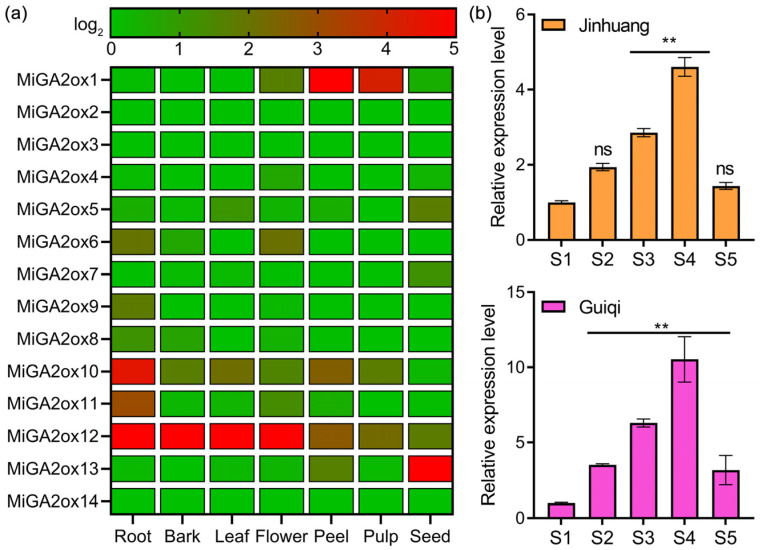
Expression profiles of *MiGA2ox* genes across various tissues and organs. (**a**) A heatmap was generated using GraphPad Prism9 software, where the (TPM + 1) values of *MiGA2ox* genes were logarithmically transformed (log2). The colors red and grey represent higher and lower relative transcript enrichment, respectively. (**b**) Quantitative PCR (qPCR) was employed to assess the expression of the *MiGA2ox12* gene in leaves of two mango cultivars, Jinhuang and Guiqi, at five different developmental stages. These stages included S1: budding stage, S2: flowering stage, S3: 3–4 weeks post-fruit setting, S4: 7–8 weeks post-fruit setting, and S5: fruit ripening stage. The symbol ** indicated a statistically significant difference in gene expression compared to stage S1 at a *p*-value of less than 0.01, while ns denoted no significant difference in gene expression relative to stage S1.

In order to explore the physiological functions of *MiGA2ox* genes, we analyzed their expression patterns in various mango tissues using RNA-seq data (Figure 5a). Notably, *MiGA2ox1*, *MiGA2ox10*, *MiGA2ox11*, and *MiGA2ox12* exhibited higher expression in special tissues, while most other genes showed relatively low expression levels across all tissues examined. Remarkably, *MiGA2ox12* demonstrated significantly high expression in four specific tissues: roots, bark, leaves, and flowers.

Additionally, we performed qPCR analysis to assess the expression of *MiGA2ox12* in leaves at five different developmental stages in two widely cultivated mango varieties in Guangxi Province (Figure 5b). The results indicated that the expression patterns of *MiGA2ox12* were similar between the two varieties, with a peak observed at stage S4 after an initial increase.

Given the prominent expression of *MiGA2ox12* in roots, bark, leaves, and flowers, as well as its spatial and temporal regulation, we hypothesize that *MiGA2ox12* may play a critical role in the growth and development of mangoes.

### 2.5. Cloning, Alignment, and Subcellular Localization of the MiGA2ox12 Gene

**Figure 6:**
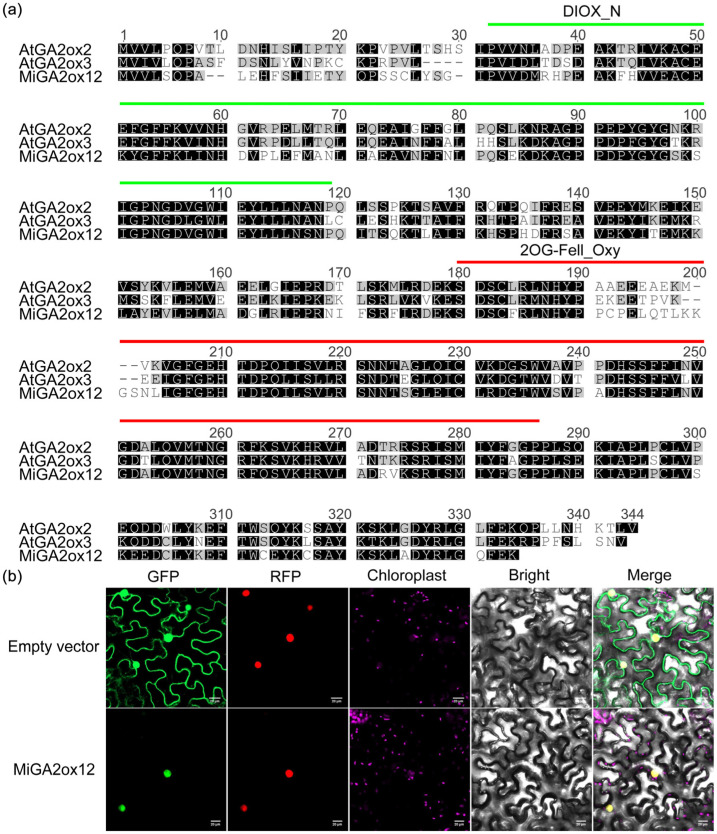
Alignment and subcellular localization analysis of MiGA2ox protein. (**a**) Protein sequences of AtGA2ox2, AtGA2ox3, and MiGA2ox12 were aligned using Clustal W. Identical residues are shaded in black, while similar residues are displayed in gray. The DIOX_N and 2OG-FeII_Oxy domains are indicated by bold lines. (**b**) For subcellular localization analysis, fusion proteins UBQ:GFP and UBQ:MiGA2ox12-GFP were transiently expressed in tobacco leaves along with the nuclear localization marker NLS-RFP and then observed using a laser scanning confocal microscope. GFP represents the protein fused with green fluorescence protein, while RFP denotes the nuclear localization protein fused with red fluorescence protein. Chloroplasts are labeled in purple, and the merged image includes signals from GFP, RFP, chloroplasts, and bright field. Scale bar = 20 µm. The experiments were performed twice.

The *MiGA2ox12* gene sequence was successfully cloned using the RACE (rapid amplification of cDNA ends) technique, with the economically significant mango variety Guiqi from the Guangxi region as the experimental material (Supplementary File S1). Comparative genomic analysis with the Alphonso cultivar revealed a single-nucleotide polymorphism (SNP) at position 129 of the protein sequence (Supplementary File S1). Sequence alignment among AtGA2ox2, AtGA2ox3, and MiGA2ox12 indicated that these three protein sequences shared conserved features, each containing the DIOX_N and 2OG-Fell_Oxy domains (Figure 6a), which are characteristic of the GA2ox family.

Subcellular localization studies conducted in tobacco leaves demonstrated that the green fluorescence of MiGA2ox12-GFP fusion protein entirely overlapped with the red fluorescence emitted by NLS-RFP (Figure 6b). The NLS serves as a nuclear localization marker, confirming that the MiGA2ox12 protein is localized within the cell nucleus.

### 2.6. Construction of Tobacco Plants with Overexpression, Knockout, and Knockout Followed by Complementation of MiGA2ox12

**Figure 7:**
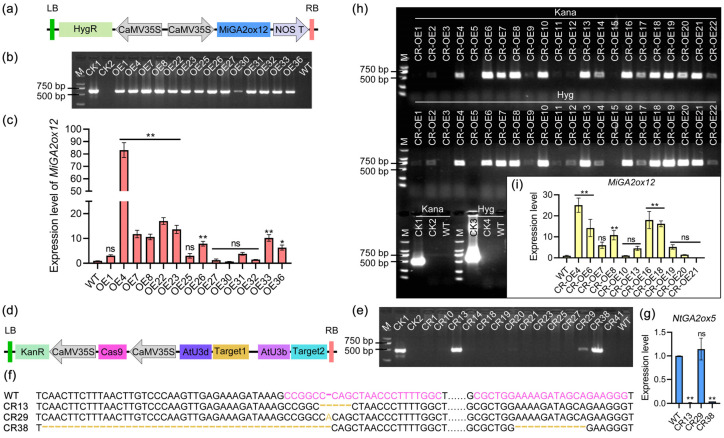
Identification of plants overexpressing *MiGA2ox12*, as well as those undergoing gene editing and subsequent replenishment. (**a**) Vector map for *MiGA2ox12* overexpression. (**b**) Identification of T0-overexpressing plants for *MiGA2ox12* using PCR cloning of the Hyg gene. CK1 served as a positive control using the overexpression vector as a template, while CK2 was the negative control utilizing ddH_2_O as a template. WT: wild-type, OE: overexpression line. (**c**) Assessment of *MiGA2ox12* gene expression levels in WT and OE lines through qPCR analysis. (**d**) Vector map related to gene editing. (**e**) Identification of T0 editing plants via PCR cloning of the Kana gene. CK1 functioned as a positive control using the editing vector as a template, and CK2 acted as a negative control using ddH_2_O as a template. CR: crisper editing line. (**f**) Comparative sequencing analysis between WT and CR lines, with pink highlights indicating the two editing targets of *NtGA2ox5* and orange marks representing the post-editing outcomes. (**g**) Expression analysis of *NtGA2ox5* gene in both WT and CR lines was conducted using qPCR. (**h**) Identification of editing and replenishing plants through PCR cloning of both Kana and Hyg genes. CK1 and CK3 were positive controls using the editing and overexpression vectors as templates, respectively. CK2 and CK4 served as negative controls using ddH_2_O as templates. CR-OE: editing and replenishing lines. (**i**) Examination of *MiGA2ox12* gene expression in WT and CR-OE plants via qPCR. “ns” indicates no significant difference from WT, while “*” and “**” denote significant differences from WT at *p* < 0.05 and 0.01 levels, respectively.

Firstly, the *MiGA2ox12* gene was overexpressed using a strong promoter 35S and hygromycin (Hyg) resistance for plant screening (Figure 7a). Fourteen positive plants were identified using the PCR cloning Hyg resistance gene sequence (Figure 7b). Among these, two plants (OE4 and OE22) showed the highest expression levels of the *MiGA2ox12* gene, which were significantly different from the wild type (WT), making them ideal candidates for overexpression lines (Figure 7c).

Next, in order to efficiently knock out the homologous gene *NbGA2ox5* of *MiGA2ox12* in tobacco, two targets were designed, and kanamycin (Kana) was used for screening resistance in plants (Figure 7d). Three positive plants were identified using PCR cloning of the Kana resistance gene sequence (Figure 7e), and sequencing analysis revealed successful editing at the target sites (Figure 7f). The expression levels of the *NbGA2ox5* gene in the gene-edited plants were significantly lower compared to the WT (Figure 7g), leading to the selection of CR13 and CR38 for further research.

In the background of the gene editing line CR38, 15 positive transgenic plants were identified to have overexpression of the *MiGA2ox12* gene through PCR cloning of the Kana and Hyg resistance gene sequence (Figure 7h). Among these, CR-OE10 and CR-0E20 were chosen as the gene editing and complementation lines for further research due to their significantly different expression levels from the WT.

### 2.7. MiGA2ox12 Inhibited Tobacco Plant Height by Degrading GA_1_ and GA_4_

**Figure 8:**
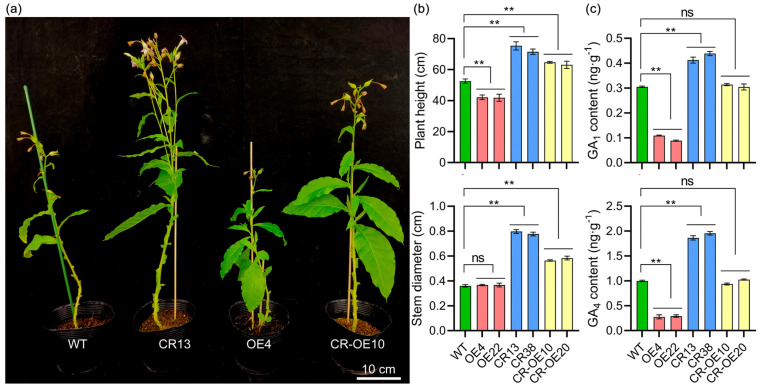
The effect of GA2ox gene on tobacco plant phenotype and GAs content. (**a**) Comparison of the phenotypes among wild-type (WT), *MiGA2ox12*-overexpressing (OE), edited (CR), and edited plus replenished plants (CR-OE). (**b**) Analysis of plant height and stem diameter. (**c**) Measurement of GA_1_ and GA_4_ content. “ns” indicates no significant difference compared to the WT, while “**” denotes a significant difference from the WT at the *p* < 0.01 level.

Representative images of the plants are displayed in Figure 8a. Overexpression of the *MiGA2ox12* gene resulted in dwarf phenotypes, while knockout of its homolog, *NtGAox5,* led to increased plant height. In a knockout background, overexpression of *MiGA2ox12* still induced dwarfism, although these plants were taller than WT plants (Figure 8b). Additionally, the stem diameter in both knockout and knockout-complemented lines was considerably larger than that of WT (Figure 8b). These findings suggest that *MiGA2ox12* functions as a negative regulator of plant height and influences stem thickness.

Given that GA2ox proteins primarily facilitate the degradation of GAs, based on phylogenetic analysis positioning MiGA2ox12 within the C_19_-GA2ox-II subfamily (Figure 3), we assessed the levels of C_19_-GAs (GA_1_ and GA_4_). Analysis revealed that the concentrations of GA_1_ and GA_4_ in overexpression lines were significantly lower compared to WT plants, while knockout lines displayed a notable increase in GA levels. No substantial changes were observed in the GA content of the knockout and complemented lines. Overall, these results indicate that the *MiGA2ox12* gene may inhibit plant height through the degradation of GA_1_ and GA_4_.

## 3. Discussion

The GA2ox family plays a crucial role in the biosynthetic and metabolic pathways of gibberellins to regulate plant height. Previous investigations have identified GA2ox family members in several species [5,6,7,12,13,20,21]. However, the presence and role of the GA2ox family in mangoes (*Mangifera indica* L.) remain uncharacterized. Given the economic and research significance of mango as a tropical fruit, exploring the function of the GA2ox family within this species is essential. In this study, bioinformatics approaches were employed to identify genes harboring the 2OG-FeII_Oxy domain in the mango genome [18], resulting in the identification of 14 *MiGA2ox* genes (Table 1). Gene structure analysis revealed that 10 out of the 14 *MiGA2ox* genes contained three exons within their coding sequence (CDS) regions (Figure 2b), consistent with prior evolutionary investigations on GA oxidase genes [22]. Furthermore, it was observed that the average length of introns in *MiGA2ox* genes exceeded that of exons, potentially introducing new functional capacities to these genes. Additionally, analysis using the MEME program revealed that all GA2ox genes possessed two shared motifs (Figure 2d).

The mango GA2ox family members were classified into three groups (C_19_-GA2ox-I, C_19_-GA2ox-II, and C_20_-GA2ox-I) (Figure 2a and Figure 3), consistent with findings in Arabidopsis, rice, peach, and other species [12,20]. These groups are named based on their substrates [20,23]. Phylogenetic analysis suggests that mango GA2ox proteins may target C20-GAs as well as C19-GAs as their substrates [6,24]. This study investigated the impact of the C_19_-GA2_OX_-II subfamily protein MiGA2ox12 on the levels of GA_1_ and GA_4_ in C19-CAs. The results showed that overexpression of the *MiGA2ox12* gene led to a decrease in the levels of GA_1_ and GA_4_ in plants (Figure 8b,c), indicating that MiGA2ox12 does indeed act on C19-CAs as substrates. Further elucidation of the function of MiGA2ox12 can be achieved through measuring the levels of the products GA_8_ and GA_34_ after the metabolism of GA_1_ and GA_4_.

Overexpression of *GA2ox* genes has been shown to lead to the degradation of highly active GAs, resulting in plant dwarfism [7,12,13,14,15]. Consistent with previous findings, overexpression of *MiGA2ox12* led to a reduction in GA_1_ and GA_4_ content and inhibited plant height (Figure 8). Consistent with expectations, the phenotypes of the knockout lines were opposite to those of the overexpression lines. However, the complementation lines did not completely restore the wild-type phenotype, possibly due to functional differences between MiGA2ox12 and NtGA2ox5. This is because even homologous genes within the same species can exhibit functional differences, such as OsGA2ox1 and OsGA2ox6 in rice exerting significantly stronger inhibitory effects on plant height compared to OsGA2ox9 [7,15,16]. However, the levels of GA_1_ and GA_4_ in the complementation lines were comparable to those in the wild type. It is speculated that NtGA2OX5 may utilize other forms of GA as substrates. This study presents a preliminary insight into the role of MiGA2ox12 in inhibiting tobacco plant height, but additional research is required to determine its impact on mango plant height. For example, it should be determined whether the inhibitory effect of overexpressed *MiGA2ox12* on plant height can be restored by the exogenous application of highly active GAs.

The binding sites for transcription factors related to plant growth and development have been identified in the *MiGA2ox* promoters (Table 2). For instance, GATA binding sites are present in all *MiGA2ox* promoters. GATA represents an important class of transcription factors that regulate plant growth, particularly those known to modulate the hormonal balance of cytokinins (CK) and GA, influencing developmental processes such as hypocotyl growth [25]. Therefore, the mechanism by which transcription factors bind to the *MiGA2ox12* promoter to activate its expression and degrade GA_1_ and GA_4_ can be further investigated. *Cis*-acting elements are essential for gene transcription and expression, so the *cis*-elements in the promoter of *MiGA2oxs* were analyzed (Figure 4). The research results indicate that the *MiGA2ox12* promoter contains a low-temperature responsive regulatory element (LTR), so the expression level of *MiGA2ox12* may be regulated by cold stress. AtGA2ox9 in Arabidopsis is induced to express under low-temperature conditions, and mutants of this gene show significantly reduced tolerance to low temperatures, suggesting that AtGA2ox9 is involved in low-temperature regulation [26]. Therefore, it can be inferred that MiGA2ox12 may participate in low-temperature regulation. Except for *MiGA2ox2*, all other promoters of MiGA2oxs contain abiotic stress response elements (Figure 4), suggesting that these genes can be induced by specific abiotic stress conditions. The expressions of genes such as *AtGA2ox7* [27,28], *PbrGA2ox1* [29], and *CsGA2ox4* [30] are upregulated under abiotic stress. Similar expressions of *GA2oxs* have also been detected in other plants. In cotton, the expression of *GhGA2ox1* in roots, stems, and leaves exhibits stronger up-regulation under salt and drought stresses [31]. In rice, several *GA2oxs* are regulated under drought conditions [32]. Recent studies on various *OsGA2ox6* mutants have demonstrated a moderate reduction in GA content alongside an increase in tolerance to abiotic and biotic stresses [33]. In addition, besides inhibiting plant height, *OsGA2ox5* and *OsGA2ox8* are also involved in positively regulating salt stress and osmotic stress, respectively [15,16,17]. Arabidopsis adapts to certain abiotic stresses primarily through short-term growth inhibition, which is achieved by inducing the expression of *GA2oxs* to reduce the bio-active GA content [27,34]. Increasing evidence suggests that the *GA2ox* genes play important roles in abiotic stresses. Similarly to OsGA2ox5, OsGA2ox6, and OsGA2ox8 [15,17], MiGA2ox12 can also be localized in the cell nucleus (Figure 6b). The localization of genes is closely related to their function; thus, MiGA2ox12 may also be involved in abiotic stress responses. Therefore, the role of MiGA2oxs in the stresses frequently encountered by mangoes remains to be explored in depth.

By analyzing the whole genome of mango, this study identified a total of 14 MiGA2ox proteins. Among them, MiGA2ox12 can degrade GA_1_ and GA_4_, leading to a decrease in the content of high active GAs and ultimately resulting in plant dwarfism. The effect of MiGA2ox12 on mango plant height, yield, and quality can be subsequently studied. If this gene does not have an impact on mango yield and quality, it is expected that it can be utilized to breed dwarf varieties and improve mango varieties.

## 4. Materials and Methods

### 4.1. Identification of MiGA2ox Members in Mango

The genome sequence of mango (*Mangifera indica* L.) was retrieved from MangoBase (https://mangobase.org/easy_gdb/tools/expression/expression_input.php, accessed on 11 May 2021). Reference protein sequences for gibberellin 2-oxidase (GA2ox) from *Arabidopsis thaliana* were acquired from TAIR (https://www.arabidopsis.org/, accessed on 12 May 2021). Candidate MiGA2ox protein sequences in mango were identified through whole-genome protein sequence alignment against the reference proteins using TBtools-II v2.131, with an E-value threshold of <10^−5^ [35]. Subsequent validation of these candidates was conducted via domain analysis (using CD-search, https://www.ncbi.nlm.nih.gov/Structure/bwrpsb/bwrpsb.cgi, accessed on 22 May 2021) [36] and motif analysis (employing MEME, http://meme-suite.org/tools/meme, accessed on 25 May 2021) [37].

### 4.2. Bioinformatics Analysis of MiGA2ox Proteins

The isoelectric points and molecular weights of MiGA2ox proteins were predicted using ExPASy (https://web.expasy.org/compute_pi/, accessed on 30 May 2021) [38]. Gene structures and chromosome locations were analyzed with TBtools [35], and the MiGA2ox proteins were subsequently renamed based on their chromosomal positions. A phylogenetic tree for GA2ox proteins from Arabidopsis, rice, and mango was constructed employing MEGA 5.0, utilizing the Maximum Likelihood method with the default parameter settings [39]. The resulting evolutionary tree in Newick format was then enhanced visually using EvolView (https://evolgenius.info//evolview-v2/#login, accessed on 10 June 2021) [40].

### 4.3. Analysis of Cis-Acting Elements and Transcription Factor Binding Sites

Promoter sequences located 2000 bp upstream of the start codon were extracted using TBtools-II v2.131 [35] and subsequently submitted to PlantCARE (http://bioinformatics.psb.ugent.be/webtools/plantcare/html/, accessed on 21 June 2021) for the prediction of *cis*-acting elements [41]. The identified elements related to growth and development, abiotic stress, and hormonal responses were cataloged. Additionally, transcription factor binding sites (TFBS) within the *MiGA2ox* promoters were predicted utilizing PlantRegMap (https://plantregmap.gao-lab.org/binding_site_prediction.php, accessed on 30 June 2021) with a threshold *p*-value set at ≤1 × 10^−4^ [42].

### 4.4. Gene Expression Analysis

Transcript abundance of *MiGA2oxs* in the root, bark, leaf, flower, peel, pulp, and seed was acquired from MangoBase (https://mangobase.org/easy_gdb/tools/expression/expression_input.php, accessed on 3 September 2021, PRJNA487154). Transcripts per million (TPM) + 1 values were subjected to log2 transformation for downstream analysis. A heatmap illustrating the expression patterns was generated using Prism9 software.

In a separate experiment, the longan cultivar “Jinhuang” and the dwarf variety “Guiqi” were cultivated at the mango resources nursery of the Guangxi Institute of Subtropical Crops. Leaves were sampled at distinct developmental stages, including budding, flowering, 3–4 weeks post-fruit setting, 7–8 weeks post-fruit setting, and fruit ripening, for quantitative real-time polymerase chain reaction (qPCR) analysis. The qPCR reagent used in this experiment was ChamQ Blue Universal SYBR qPCR Master Mix (Q312, Vazyme, Nanjing, China). The instrument for qPCR was produced by Applied Biosystems (Stetone Plus, Waltham, MA, USA). Primers were designed using Beacon Designer v8.14, with mango *Actin* serving as the reference gene. Data analysis was conducted utilizing the 7300 System software and the 2^−ΔΔCt^ method [42]. Details of the primers can be found in Supplementary Table S1.

### 4.5. Cloning of MiGA2ox12 Gene via Rapid-Amplification of cDNA Ends (RACE) Technique

In this experiment, we utilized the Guiqi mango variety, extensively cultivated in the Guangxi region, as a template to clone the *MiGA2ox12* gene through rapid amplification of cDNA ends (RACE) technology [43]. The cDNA sequence of the *MiGA2ox12* gene, as recorded in GenBank (LOC123192218), served as the basis for designing specific primers. These primers were formulated conducting an alignment with the DNAMANv9.0 software to identify conserved regions, resulting in the generation of an intermediate fragment. Subsequent to sequencing this fragment, we developed primers for both 3′ RACE and 5′ RACE to acquire the respective 3′ and 5′ end sequences for further sequencing. The complete sequence of the *MiGA2ox12* gene was assembled using DNAMAN by integrating the 5′ and 3′ end sequences along with the intermediate fragment, followed by a comparative analysis in the NCBI database. Details regarding the primers can be found in Supplementary Table S1.

### 4.6. Subcellular Localization of MiGA2ox12 in Tobacco Leaves

The coding sequence of *MiGA2ox12* was amplified by PCR and subsequently cloned, resulting in a fusion with green fluorescent protein (GFP) at the C-terminus, under the control of the UBQ promoter. The recombinant plasmids were confirmed through enzymatic digestion and sequencing analysis. In academic research, a nuclear localization sequence (NLS) fused with a red fluorescent protein (RFP) is commonly employed as a marker for nuclear localization. Thereafter, the plasmids containing GFP and RFP were introduced into *Agrobacterium tumefaciens* strain GV3101, respectively. The transformed GV3101 cultures were cultivated until reaching an optical density of approximately 1.0 at 600 nm (OD600), followed by dilution in a buffer solution (10 mM MgCl_2_ and 150 µM acetosyringone) to an OD600 of 0.2. Equal volumes of GFP and RFP mixtures were combined, and the resulting bacterial suspension was incubated at room temperature for one hour. Subsequently, this suspension was delivered into the abaxial side of tobacco leaves using a 1 mL syringe. After injection, the plants were kept in darkness for a duration of two days.

Green fluorescence emitted from tobacco leaves was excited with a 488 nm laser with emission captured between 495–550 nm. Red fluorescent was stimulated at 554 nm, and the emitted light was detected in the range of 580–630 nm. Chloroplast fluorescence was excited using a 470 nm laser and recorded within the same range as the green fluorescence (495–550 nm). Image acquisition was performed utilizing an OLYMPUS FV10-MCPUS confocal laser scanning microscope (Olympus, Tokyo, Japan). Image processing was conducted with the FV10-ASW 3.0 software and Adobe Photoshop CS4 version 11.0.2.

### 4.7. Construction of Gene Overexpression, Gene Knockout, and Gene Knockout with Complementation in Tobacco

For gene overexpression, the pCAMBIA1301s vector was linearized via *Sal* I/*Pml* I digestion (R0138V/R0532V, New England Biolabs, Ipswich, MA, USA), followed by ligation with the *MiGA2ox12* coding sequence using T4 DNA ligase (DNA Ligation Kit Ver.2.1, 6022Q, Takara, Shiga, Japan). For gene editing, we designed sgRNA targets based on the NtGA2ox5 sequence and amplification primers according to the target sequences (Table S1). The amplified products and the pYLCRISPR/Cas9 vector were digested with the *Bas* I enzyme and then connected using T4 ligase. Successful positive clones were confirmed through Sanger sequencing, resulting in the construction of the pCAMBIA1301s-MiGA2ox12 vector for plant studies. This vector was subsequently introduced into Agrobacterium strain GV3101 to facilitate agrobacterium-mediated transformation in tobacco plants.

In tobacco genetic transformation experiments, four main steps are required: co-culture, callus induction, resistant bud induction, and rooting induction. Briefly, sterile young tobacco leaves were firstly immersed in a bacterial solution for 20 min, with gentle shaking every 2–3 min. The leaves were then placed with their abaxial surfaces on co-cultivation medium Tob2 and culture for three days at 22 °C under weak light conditions. They were then transferred to selection medium Tob3 for further cultivation until callus tissue generation. The conditions were set at 28 °C with a light photoperiod of 16 h of light and 8 h of dark, providing a light intensity of 2500 Lux. The callus tissue was then excised and transferred to elongation medium Tob4 and cultivated. The shoots grew to 1–2 cm. The medium was changed every three weeks during this period. Subsequently, they were transferred to the rooting medium Tob 5 for root induction. The cultivation conditions during this stage included 25 °C, 16 h of light, and 8 h of dark, maintaining a light intensity of 2500 Lux. The composition of Tob2 was 4.43 g/L MS (M519, Phytotech, Lenexa, KS, USA), 30 g/L sucrose (1002141933, SCR, Peking, China), 8 g/L agar (A8190, Solarbio, Peking, China), pH 5.4, 1 mg/L 6-Benzylaminopurine (BA, B3408, Sigma, Kawasaki, Japan), 0.2 mg/L α-Naphthaleneacetic acid (NAA, N0640, Sigma, Kawasaki, Japan), and 100 μM Acetylsyringone (As, D124406, Sigma, Kawasaki, Japan). The composition of Tob3 was 4.43 g/L MS, 30 g/L sucrose, 8 g/L agar, pH 5.8, 1 mg/L BA, 0.2 mg/L NAA, and 500 mg/L carbenicillin (Carb) supplemented with appropriate antibiotics. For overexpression-based plant resistance selection, hygromycin (Hgy, 10843555001, Roche, Basel, Switzerland) was applied at a concentration of 15 mg/L. Conversely, knockout-based selection utilized kanamycin (Kana, K8020, Solarbio) at a concentration of 50 mg/L. For combined gene knockout and complementation approaches, the suggested concentrations remained 15 mg/L for Hgy and 50 mg/L for Kana. The composition of Tob4 was 4.43 g/L MS, 30 g/L sucrose, 8 g/L agar, and pH 5.8. The composition of Tob5 was 4.43 g/L MS, 30 g/L sucrose, 8 g/L agar, pH 5.8, and 0.1 mg/L NAA. Transgenic shoots were verified through PCR to confirm the presence of the antibiotic resistance genes (primers listed in Table S1).

### 4.8. Phenotypic Characteristics and Gibberellin Assay

For gibberellin quantification, approximately 100 mg of each plant sample was combined with 1 mL of pre-cooled 50% aqueous solution. The mixture underwent ultrasonication at 4 °C for three minutes, followed by a static extraction period of thirty minutes. Subsequently, the sample was centrifuged at 12,000 rpm for ten minutes, and the supernatant was collected and purified using reverse-phase solid-phase extraction (RP-SPE) columns.

The extracted samples were analyzed utilizing a UPLC-Orbitrap mass spectrometry system (UPLC, Vanquish; MS, QE). The analytical conditions were meticulously optimized: the UPLC column was Waters HSS T3 (50 mm × 2.1 mm, 1.8 μm). The mobile phase comprised solvent A (ultrapure water supplemented with 0.1% acetic acid) and solvent B (acetonitrile with 0.1% acetic acid). The flow rate was maintained at 0.3 mL/min, with the column temperature set to 40 °C and an injection volume of 2 μL.

The elution gradient was programmed with specific volume ratios of phase A to phase B at the following time points: 0 min (85:15), 0.5 min (85:15), 1.5 min (10:90), 3.0 min (10:90), 3.1 min (85:15), and 5.0 min (85:15). High-resolution mass spectrometry (HRMS) data were acquired using a Q-Exactive hybrid Q-Orbitrap mass spectrometer (Thermo Fisher Scientific). The electrospray ionization (ESI) source parameters were set as follows: sheath gas flow rate of 40 arb, auxiliary gas flow rate of 10 arb, ion spray voltage of −2800 V, temperature of 350 °C, and ion transfer tube temperature of 320 °C.

Data collection was conducted on the Q-Exactive platform and subsequently processed with Xcalibur 4.1 (Thermo Scientific) and TraceFinder™ 4.1 Clinical (Thermo Fisher Scientific, Waltham, MA, USA). Standard curves were generated using TraceFinder software, enabling absolute quantification of compounds via the external standard method.

### 4.9. Statistical Analysis

All experiments were conducted in triplicate without specific instructions. Statistical significance was assessed using one-way ANOVA, conducted with Prism9 software (GraphPad, San Diego, CA, USA). Asterisks in the figures denote significant differences, with * *p* < 0.05, ** *p* < 0.01, *** *p* < 0.001.

## 5. Conclusions

The identification and characterization of the GA2ox gene family in mango have resulted in the discovery of 14 *MiGA2ox* genes, which have been classified into C_20_-GA2ox-I, C_19_-GA2ox-I, and C_19_-GA2ox-II. The expression of *MiGA2ox* genes exhibits significant spatiotemporal specificity. Therefore, exploring the function of each gene in mango growth and development is of great importance. Functional studies on *MiGA2ox12*, which is highly expressed in multiple tissues, indicate that *MiGA2ox12* negatively regulates plant height by degradation of GA_1_ and GA_4_. This research provides a scientific foundation for a comprehensive understanding of the *GA2ox* gene family in mango and offers new insights for the genetic improvement of plant height in mangoes.

## Figures and Tables

**Table 1:** Characteristics of the putative mango *MiGA2ox* genes.

Name	Chromosome Location	Gene ID	Chr	Strand	Protein ID	CDS	AA	PI	MW (kDa)
*MiGA2ox1*	24,503,011–24,504,683	LOC123214473	Chr1	+	XP_044490187.1	999	332	6.17	37.58
*MiGA2ox2*	27,312,023–27,313,741	LOC123193387	Chr1	−	XP_044462323.1	588	195	5.31	39.34
*MiGA2ox3*	27,322,479–27,324,300	LOC123228317	Chr1	−	XP_044509611.1	1005	334	8.69	21.99
*MiGA2ox4*	17,046,678–17,048,639	LOC123209695	Chr2	+	XP_044483758.1	987	328	7.61	37.77
*MiGA2ox5*	4,943,584–4,945,495	LOC123213537	Chr4	−	XP_044488928.1	1029	342	6.13	39.96
*MiGA2ox6*	17,570,772–17,573,157	LOC123217611	Chr5	+	XP_044494640.1	1011	336	5.59	37.23
*MiGA2ox7*	1,267,189–1,268,697	LOC123218572	Chr6	−	XP_044495998.1	1041	346	5.33	38.38
*MiGA2ox8*	20,172,193–20,175,085	LOC123221737	Chr7	−	XP_044500573.1	996	331	7.63	37.37
*MiGA2ox9*	2,822,944–2,825,112	LOC123224430	Chr8	−	XP_044504019.1	1002	333	7.7	37.67
*MiGA2ox10*	12,248,437–12,250,210	LOC123225356	Chr9	+	XP_044505227.1	990	329	6.38	39.16
*MiGA2ox11*	12,739,363-12,744,177	LOC123192364	Chr12	+	XP_044460826.1	1038	345	5.87	36.94
					XP_044460827.1	1038	345	5.87	36.94
					XP_044460828.1	1038	345	5.87	36.94
*MiGA2ox12*	15,118,064–15,119,695	LOC123192218	Chr12	+	XP_044460627.1	996	331	6.76	37.39
*MiGA2ox13*	8,371,702–8,373,428	LOC123201642	Chr18	−	XP_044473167.1	1020	339	6.47	37.73
					XP_044473168.1	1005	334	7.62	37.22
*MiGA2ox14*	11,375,751–11,380,933	LOC123202384	Chr18	−	XP_044474238.1	1044	347	5.4	38.26
					XP_044474239.1	1044	347	5.4	38.26

**Table 2:** Types and quantities of transcription factor binding sites (TFBSs) identified within the promoters of *MiGA2ox* genes.

	*MiGA2ox1*	*MiGA2ox2*	*MiGA2ox3*	*MiGA2ox4*	*MiGA2ox5*	*MiGA2ox6*	*MiGA2ox7*	*MiGA2ox8*	*MiGA2ox9*	*MiGA2ox10*	*MiGA2ox11*	*MiGA2ox12*	*MiGA2ox13*	*MiGA2ox14*
AP2	9	6	3	7	12	2	8	14	14	4	5	3	5	19
ARF	10	4	8	4	1	4	1	5	1	1	7	0	4	2
ARR-B	7	0	1	1	0	1	2	0	0	0	3	0	0	0
B3	8	7	2	25	5	6	16	17	6	8	0	6	3	24
BBR-BPC	20	27	6	2	102	16	13	34	23	4	20	39	2	100
BES1	2	0	2	0	0	12	0	0	1	0	0	0	9	2
bHLH	8	12	6	1	22	35	0	4	34	7	48	12	35	10
bZIP	20	22	13	2	6	22	11	1	27	6	49	5	26	27
C2H2	44	33	27	31	79	11	6	36	31	15	10	16	15	17
CAMTA	1	0	6	0	0	0	0	0	5	0	3	0	1	1
C3H	0	1	0	0	1	1	2	4	3	2	0	0	0	0
CPP	5	5	7	3	5	8	7	4	12	7	2	7	20	2
Dof	23	34	28	25	85	55	10	97	60	26	20	40	10	34
E2F/DP	12	0	4	2	6	3	1	0	2	5	1	2	1	0
EIL	0	3	0	0	4	0	1	0	0	0	1	0	0	1
ERF	347	432	29	60	77	83	1	4	64	67	70	1	113	36
FAR1	0	0	6	0	0	1	0	0	4	0	2	0	0	1
G2-like	25	0	1	13	14	10	5	1	4	8	3	10	2	1
GATA	4	17	14	6	17	10	3	0	11	12	10	3	9	2
GeBP	4	5	2	7	0	2	0	0	3	1	3	0	1	0
GRAS	5	4	5	2	16	2	6	7	6	3	5	8	1	17
GRF	0	1	2	0	0	1	0	0	0	0	1	1	0	0
HD-ZIP	2	0	10	11	21	13	35	7	3	23	27	12	4	11
HSF	21	2	1	6	2	0	11	0	1	10	7	15	4	2
LBD	47	11	0	3	4	2	1	2	5	2	0	0	6	1
LFY	0	0	2	2	0	0	0	0	2	2	0	0	0	0
MIKC_MADS	6	54	19	20	41	10	4	20	15	9	20	17	4	32
MYB	53	16	48	15	15	45	19	29	40	25	16	6	40	21
MYB_related	12	2	19	22	8	6	5	20	4	8	33	9	7	11
NAC	4	27	16	10	16	2	6	9	5	11	34	2	3	2
NF-YB	1	0	0	0	1	0	0	1	1	0	0	0	1	0
Nin-like	8	7	3	1	5	1	1	1	7	0	4	3	3	1
RAV	0	9	1	0	1	0	0	1	1	1	0	0	3	1
S1Fa-like	3	0	0	0	1	0	1	0	0	1	0	2	1	0
SBP	6	6	1	0	0	0	2	3	0	0	3	1	8	0
SRS	4	0	3	1	1	0	0	2	0	0	2	1	1	0
TCP	19	6	0	4	21	22	16	4	1	0	13	1	35	9
Trihelix	20	8	6	6	0	7	6	8	9	2	12	1	3	1
VOZ	0	0	0	0	0	0	0	0	1	1	0	0	0	0
WOX	1	0	1	4	3	0	0	0	1	2	3	1	1	7
WRKY	0	83	33	66	26	31	2	37	4	1	8	2	1	62
YABBY	0	0	1	0	0	3	4	0	0	0	1	0	0	1
ZF-HD	0	0	2	5	1	2	6	1	5	8	6	5	3	4
Number of TFBS type	33	28	36	31	32	32	31	28	36	31	34	29	35	32
Sum of TFBS numbers	761	844	338	367	619	429	212	373	416	282	452	231	385	462

## Data Availability

Data is contained within the article and Supplementary Materials.

## Supplementary Materials

The following supporting information can be downloaded at: https://www.mdpi.com/article/10.3390/ijms252212109/s1.
