# Supplementary material for: Over Expression of Mango MiGA2ox12 in Tobacco Reduced Plant Height by Reducing GA1 and GA4 Content
Source: Int J Mol Sci. 2024 Nov 11;25(22):12109. doi: 10.3390/ijms252212109 (PMC11594832; doi:10.3390/ijms252212109)
Supplement: Supplementary file 1 [file ijms-25-12109-s001.zip › ijms-3278042-File S1.pdf]

RACE Clone Sequencing Results：

```
1       ACATGGGGGA GAGTGGTACT AGTATAGGGC GATTGGGCCC GACGTCGCAT
51      GCTCCCGGCC GCCATGGCGG CCGCGGGAAT TCGATTAATG GCGATGTTGG
101     ATGGATTGAA TACCTCCTCT TCACCGCCAA TCAAGATTCT AATCTCCAAC
151     GATTCGTTTA TCCTCTCGGA AAAAACCCGG AAAAGTTTCG GTATAAACCT
201     GCAGAAAAAA TCATTCCTTT TTTATTTTCG TTTGCATAGC TGTGTTTTAT
251     AGTCTTGTTT GGTGTTGCTG CAGTCGTGGC AATTTCTGCC CGTTCTTTCC
301     CTTCCTCAAT GGCATAAATA CCAGGCAGAC ACACAAAGCT TCTTTATAGT
351     TAGCAACGCC TCTCTCTGCC TCTGTCTTCA TGCATCAGCA GCAAATCCTC
401     TCATTTGCCC ATATAAACCC TTCAATCTCT TCAGACCCTT CTCAATTCTC
451     ACCTTCTCCT CTTTTCCACT CTCAATTTCT CTCTCTGTCT CTCTCTGTGT
501     TTTGTCAGAA AAAACTCGGC CACCATGGTG GTTCTGTCAC AACCAGCATT
551     AGAACATTTC TCTATAATCG AAACTTACCA GCCTTCAAGC TGCTTATACT
601     CAGGAATTCC AGTTGTAGAC ATGAGACACC CTGAAGCCAA GTTCCATGTA
651     GTGGAAGCCT GTGAAAAATA CGGCTTCTTC AAGCTCATTA ACCATGATGT
701     TCCGTTGGAG TTCATGGCCA ATTTAGAAGC CGAAGCTGTC AACTTCTTTA
751     ACCTCCCTCA GTCTGAGAAA GACAAAGCTG GACCCCCTGA CCCTTATGGC
801     TATGGCAGCA AAAGCATTGG CCCCAATGGT GATGTTGGTT GGATTGAATA
851     TCTCCTCCTC AACTCCAACC CTCAAATCAC TTCACAAAAA ACTCTCGCCA
901     TTTTCAAACA CAGCCCTCAT GATTTCCGGA GTGCTGTGGA GAAGTACATA
951     ACAGAAATGA AGAAACTTGC ATATGAAGTT CTTGAATTAA TGGCCGATGG
1001    GCTAAGAATA GAGCCAAGGA ACATTTTCAG TAGATTCATA AGGGATGAAA
1051    AAAGTGACTC CTGTTTCAGG CTGAACCACT ACCCACCATG TCCAGAGCTT
1101    CAAACATTGA AGAAAGGAAG CAATTTGATT GGGTTCGGAG AACACACAGA
1151    CCCTCAGATT CTTTCTGTTC TAAGATCTAA CAATACTTCA GGACTTGAAA
1201    TTTGCTTAAG AGATGGCACT TGGGTTTCTG TCCCTGCTGA TCATTCTTCC
1251    TTTTTCCTCA ATGTTGGTGA TGCTCTGCAG GTAATGACTA ATGGGAGATT
1301    CCAAAGTGTG AAGCACAGAG TGTTGGCTGA CAGAGTGAAA TCAAGAATTT
1351    CAATGATATA TTTTGGAGGG CCACCATTGA ATGAAAAGAT TGCACCTTTG
1401    CCATGCCTAG TTTCAAAAGA AGAAGATTGC TTGTACAAGG AGTTCACTTG
1451    GTGTGAATAC AAGTGCTCTG CCTATAAGTC CAAGTTGGCT GATTATAGGC
1501    TTGGGCAGTT TGAGAAATGA AAACCAAAAG GACACTTCCG TAATTTAGCA
1551    AAAATTAAAC TAGCCATACA TGGAGAAGAA TATAAAATAG AGCCTCAACT
1601    TCTCTTGTTT ATTTATCAAC AACTCTGTAC TAGTTAACTG CAGCTACAAT
1651    GTTATTATCC ACTAGATCTT GATCATGTTA AAATAAATTA TAATTTTCAA
1701    CTTATTTTCA TATTTTATTT TATGTTAACC ACAGTTTCAA TTCCCCCTTT
1751    GGGTTTCTCA TTGTCCTGGA ATTTAGGCAT TCAATTTGAG TATTTGACTT
1801    GGCATACATG CACGTCATTC TTATCTATAA CTATCGAATA TAGCTGCCAT
1851    CAAAAGCTTT TGCCAGTACC CAAGCATGAA ATGTTTTTTC CTATCTGTAT
1901    TGATGATCAA TAGAGTTGAA TTACTGTTCG TTAATGGAGA TTTCTGTGTT
1951    GGGTGGTTGA TTATGCAGGT GCTGACAAAT GGAAGATTTA AGAGCGTAAG
2001    GCAAAATGTT GGAGACTCAT TACAGGTATA CAATTTGTAC AGTTTTTACC
2051    AAAAAAAAAA AAAAAAAAAA AAAAA
```

RACE blast result in NCBI database

Download GenPept Graphics

gibberellin 2-beta-dioxygenase-like [Mangifera indica]

Sequence ID: XP_044460627.1 Length: 331 Number of Matches: 1

Range 1: 1 to 331 GenPept Graphics ▼ Next Match ▲ Previous Match

Score	Expect	Method	Identities	Positives	Gaps	Frame
690 bits(1780)	0.0	Compositional matrix adjust.	330/331(99%)	330/331(99%)	0/331(0%)	+3

```
Query  525   MVVLSQPALEHFSIIETYQPSSCLYSGIPVVDMRHPEAKFHVVEACEKYGFFKLINHDVP  704
             MVVLSQPALEHFSIIETYQPSSCLYSGIPVVDMRHPEAKFHVVEACEKYGFFKLINHDVP
Sbjct  1     MVVLSQPALEHFSIIETYQPSSCLYSGIPVVDMRHPEAKFHVVEACEKYGFFKLINHDVP  60

Query  705   LEFMANLEAEAVNFFNLPQSEKDKAGPPDPYGYGSKSIGPNGDVGWIEYLLLNSNPQITS  884
             LEFMANLEAEAVNFFNLPQSEKDKAGPPDPYGYGSKSIGPNGDVGWIEYLLLNSNPQITS
Sbjct  61    LEFMANLEAEAVNFFNLPQSEKDKAGPPDPYGYGSKSIGPNGDVGWIEYLLLNSNPQITS  120

Query  885   QKTLAIFKHSPHDFRSAVEKYITEMKKLAYEVLELMADGLRIEPRNIFSRFIRDEKSDSC  1064
             QKTLAIFK SPHDFRSAVEKYITEMKKLAYEVLELMADGLRIEPRNIFSRFIRDEKSDSC
Sbjct  121   QKTLAIFKQSPHDFRSAVEKYITEMKKLAYEVLELMADGLRIEPRNIFSRFIRDEKSDSC  180

Query  1065  FRLNHYPPCPELQTLKKGSNLIGFGEHTDPQILSVLRSNNTSGLEICLRDGTWVSVPADH  1244
             FRLNHYPPCPELQTLKKGSNLIGFGEHTDPQILSVLRSNNTSGLEICLRDGTWVSVPADH
Sbjct  181   FRLNHYPPCPELQTLKKGSNLIGFGEHTDPQILSVLRSNNTSGLEICLRDGTWVSVPADH  240

Query  1245  SSFFLNVGDALQVMTNGRFQSVKHRVLADRVKSRISMIYFGGPPLNEKIAPLPCLVSKEE  1424
             SSFFLNVGDALQVMTNGRFQSVKHRVLADRVKSRISMIYFGGPPLNEKIAPLPCLVSKEE
Sbjct  241   SSFFLNVGDALQVMTNGRFQSVKHRVLADRVKSRISMIYFGGPPLNEKIAPLPCLVSKEE  300

Query  1425  DCLYKEFTWCEYKCSAYKSKLADYRLGQFEK  1517
             DCLYKEFTWCEYKCSAYKSKLADYRLGQFEK
Sbjct  301   DCLYKEFTWCEYKCSAYKSKLADYRLGQFEK  331
```
